# Trends in Parkinson’s disease medication prescribing patterns in the UK: An interrupted time series analysis (2019–2024)

**DOI:** 10.1371/journal.pone.0324999

**Published:** 2025-05-23

**Authors:** Khalid Orayj

**Affiliations:** Clinical Pharmacy Department, School of Pharmacy, King Khalid University, Abha, Saudi Arabia; University of Toledo College of Medicine: The University of Toledo College of Medicine and Life Sciences, UNITED STATES OF AMERICA

## Abstract

This study aimed to examine prescribing trends for Parkinson’s disease (PD) medications in the United Kingdom from 2019 to 2024, focusing on the impact of guidelines from the American Academy of Neurology (AAN) and the National Institute for Health and Care Excellence (NICE) on the use of levodopa and dopamine agonists (DAs). A repeated cross-sectional design was employed, using publicly available data to assess prescribing patterns across the four UK countries. An interrupted time series analysis with linear regression was performed to identify trends, comparing regions with England as the reference point. Levodopa remained the most prescribed PD medication across all UK regions, as revealed by the analysis. In England, levodopa prescriptions increased significantly after the introduction of AAN guidelines, while other regions displayed more stable trends. Northern Ireland exhibited a distinct pattern, with DAs prescribed more frequently than levodopa. The findings also indicated that Scotland and Wales were less responsive to AAN guidance. This study highlights the influence of clinical guidelines on PD prescribing practices in the UK, with regional variations suggesting possible demographic or healthcare system factors. Further research is required to understand these disparities and their implications for PD management.

## Introduction

Parkinson’s disease (PD) is a progressive neurodegenerative disorder characterized by the degeneration of dopaminergic neurons, leading to motor and non-motor symptoms that significantly impact patients’ quality of life [1]. PD is a significant public health concern, with an estimated prevalence of 1% in people over the age of 60 and 3% in those over 80 [2]. As the global population ages, PD continues to present a growing health challenge, necessitating ongoing research into its epidemiology and treatment options.

In the UK, efforts to assess the epidemiology of PD have led to several important studies. One cohort study, conducted between 2006 and 2016, analyzed individuals aged 50 years and older using a large UK primary care database. This study calculated PD incidence rates based on different case definitions, accounting for factors such as diagnosis, symptoms, and treatment. The findings revealed that the incidence of PD remained stable, with no significant changes in underlying risk factors during this period [3]. This suggests that despite advancements in diagnostic techniques and increased awareness, the incidence rate of PD has remained stable in the UK and globally over the past decade [4,5]. A comprehensive review of UK prevalence studies, conducted between 1961 and 2007, highlights the complexities involved in accurately measuring PD prevalence. Differences in methodology for case ascertainment and diagnosis led to a wide range of prevalence estimates, varying from 105 to 168 per 100,000 individuals. Despite these variations, the review found no clear trend of increasing or decreasing PD prevalence during the studied period. Additionally, there were no significant differences observed between rural and urban populations, and the impact of ethnicity on PD prevalence remains an underexplored area [6]. These findings highlight the need for more focused research to address potential demographic influences on PD epidemiology.

The pharmacological treatment landscape for PD has evolved significantly over the past 30 years. Initially, treatment options were limited to levodopa and anticholinergics. However, recent decades have seen the introduction of non-ergot dopamine agonists (DAs), monoamine oxidase-B (MAO-B) inhibitors, and catechol-O-methyl transferase (COMT) inhibitors. Although these newer therapies provide additional options for managing PD symptoms, their long-term efficacy and safety have been extensively studied through clinical trials and post-marketing surveillance. A review by the American Academy of Neurology (AAN) in 2006 concluded that DAs, MAO-B inhibitors, and levodopa do not provide disease-modifying properties, reinforcing the emphasis on symptomatic management as the primary treatment goal for PD [7].

DAs were initially introduced to reduce the motor complications associated with long-term levodopa use, such as dyskinesia. Several clinical trials conducted between 1989 and 2006 compared levodopa to various DAs, including bromocriptine, ropinirole, and pramipexole. These trials found that starting treatment with DAs delayed the onset of dyskinesia or motor fluctuations, leading to guidelines recommending the use of DAs as a first-line therapy for younger patients [8,9]. However, subsequent studies, such as the PD-MED trial, showed that early use of levodopa led to better long-term quality of life (QoL) outcomes compared to DAs and MAO-B inhibitors [10].

Despite these findings, there remains an ongoing debate over the most appropriate first-line therapy for newly diagnosed PD patients. The National Institute for Health and Care Excellence (NICE) in the UK recommends initiating levodopa treatment for patients whose motor symptoms significantly impact their QoL, while DAs and MAO-B inhibitors are suggested as alternatives for patients with less severe symptoms [11]. Interestingly, despite these established guidelines, studies on prescribing patterns in the UK have shown varying trends. For instance, a study using the UK Clinical Practice Research Datalink (CPRD) found a relatively low rate of levodopa being prescribed as the initial therapy between 2004 and 2015, with only 29% of patients starting treatment with levodopa [12]. On the other hand, a population-based study in Wales indicated a significant shift toward levodopa as the preferred first-line therapy between 2000 and 2016 [13].

Since the release of the 2017 NICE guidelines, no major studies have reassessed PD prescribing trends in the UK. In contrast, the 2021 update by the American Academy of Neurology (AAN) took a clearer stance, recommending levodopa as the preferred first-line treatment for early-stage PD regardless of quality-of-life impact. The AAN also advised limiting DAs use to younger patients at higher risk of dyskinesia, and avoiding them in older individuals or those with cognitive or behavioral vulnerabilities [14]. This divergence underscores the need to examine whether such guidelines have influenced real-world prescribing in the UK, particularly following the clearer 2021 AAN recommendations.

This study aims to address the gap by applying an interrupted time series (ITS) segmented regression design—an approach not previously used to evaluate PD medication prescribing across all four UK countries—to analyze prescribing patterns from 2019 to 2024, with a particular focus on the impact of the American Academy of Neurology (AAN) recommendations. By examining data on levodopa and other PD medications, the study seeks to determine whether the AAN guidelines led to an increase in levodopa prescriptions and to provide a comprehensive overview of prescribing trends during this period. This method, supported by robust diagnostics, also offers insights into the influence of clinical guidelines on prescribing behavior and highlights regional differences. Investigating these regional patterns is critical, as they may reflect variations in healthcare access, prescriber preferences, and population characteristics that influence clinical outcomes and guideline implementation.

## Materials and methods

### Study design

This study employed a repeated cross-sectional design to analyze the prescribing patterns of PD medications across the United Kingdom from July 2019 to May 2024, selected due to comprehensive data availability from all four UK countries. The analysis encompassed all PD medications, which were categorized into six main groups. The list of medications and their British National Formulary (BNF) codes can be found in Table 1. Any medications without recorded prescriptions were excluded from the analysis, with BNF codes used to identify the relevant drugs.

**Table 1:** Parkinson’s disease medications and corresponding BNF codes.

PD medication category		PD medication and BNF codes
Anticholinergics		Benzatropine mesilate: 0409020E0
		Orphenadrine hydrochloride: 0409020N0
		Procyclidine hydrochloride: 0409020S0
		Trihexyphenidyl hydrochloride: 0409020C0
Dopamine Agonists (DAs)	Ergot DAs	Cabergoline: 0409010U0
		Pergolide mesilate: 0409010P0
	Non-ergot DAs	Apomorphine: 0409010A
		Pramipexole: 0409010W0
		Ropinirole hydrochloride: 0409010H0
		Rotigotine: 0409010Z0
Levodopa		Co-beneldopa (Benserazide/levodopa): 0409010K0, Co-careldopa (Carbidopa/levodopa): 0409010N0, Levodopa/carbidopa/entacapone: 0409010X0
MAO-B inhibitors		Rasagiline mesilate: 0409010Y0
		Safinamide: 0409010AA
		Selegiline hydrochloride: 0409010T0
COMT inhibitors		Entacapone: 0409010V0, Levodopa/carbidopa/entacapone: 0409010X0
		Opicapone: 0409010AB
		Tolcapone: 0409010S0
Amantadine		Amantadine hydrochloride: 0409010B0

Data for this study were gathered from publicly available sources. Prescribing data for England were obtained from OpenPrescribing.net [15], for Wales from the NHS Wales Shared Services Partnership’s Prescribing Data Extracts [16], for Scotland from Public Health Scotland’s Monthly Prescribing Activity [17], and for Northern Ireland from the GP Prescribing Data on Open Data Northern Ireland [18]. All the data used in this research are openly accessible under the Open Government License (OGL) and did not require ethical approval. These data were pre-aggregated by month and region and do not include any patient-level identifiers or sensitive information, thereby ensuring full compliance with privacy and confidentiality standards. All monthly prescribing Excel files were downloaded and grouped by region and time period to ensure consistency and accuracy in the analysis. Since no data were missing, the dataset was directly prepared for analysis without the need for further data cleaning or imputation, ensuring the integrity and robustness of the dataset used in the study.

In the UK, while most Parkinson’s patients are managed by Care of the Elderly (COTE) physicians, neurologists, and Parkinson’s Disease Nurse Specialists (PDNS), these specialists provide general practitioners (GPs) with recommendations regarding the initiation, titration, or modification of PD medications [19]. Consequently, the majority of PD prescriptions are expected to be captured within GP prescribing data. The study focused exclusively on prescriptions issued by GPs in community settings, excluding those from hospitals or other healthcare facilities.

### Prevalence calculation

To estimate the prevalence of PD medication prescriptions, the number of prescriptions each month was divided by the corresponding country’s population for that month, then multiplied by 100,000 to calculate a standardized prescription rate per 100,000 people. Population figures were sourced from official government websites [20]. However, due to the absence of population data for Scotland and Northern Ireland for 2023, and for all four countries in 2024, population figures for these periods were projected using the growth rate from the previous year. Given the short projection period and the relatively stable year-on-year trends in UK population data, this was considered a reasonable and practical approach. Alternative methods, such as linear extrapolation or using national projections, were also considered but would likely have produced similar results over such a limited timeframe.

### Statistical analysis

In this study, an interrupted time series (ITS) analysis with a linear regression model using the backward elimination method was employed to assess PD medication prescribing patterns in the UK from July 2019 to May 2024. ITS segmented regression design was chosen for this study because it is well-suited to evaluate the impact of interventions or policy changes over time. This approach enables the distinction between pre-intervention trends and post-intervention changes in level (an immediate shift in prescribing rates) and trend (a change in the rate of increase or decrease over time), providing a robust framework to assess the effect of the AAN guideline introduction on prescribing patterns. This design is particularly effective at detecting abrupt changes in trends but may be less sensitive to gradual shifts over time. The analysis was conducted using SPSS version 28. The model incorporated various factors, including prescribing trends, level changes, and country comparisons, with England serving as the reference point through an interaction term. To enhance the explanatory power of the model, all major medications were included, and the final model retained variables even if trends or levels were removed, as their exclusion would indicate no significant effect. Additionally, for each PD medication model, the effect of other PD medications prescribing patterns was examined as a potential factor, to account for any interdependencies among medications. The ergot DAs model was not performed because of very low prescribing rates, and instead, these were combined with non-ergot DAs into a category labeled “All DAs”.

Given the complexity of the data, variance inflation factors (VIFs) were used to assess multicollinearity, with a range of 5–10 considered acceptable [21]. If multicollinearity was detected (VIF > 10), the affected variable was removed, prioritizing the retention of trend or level, as these were key variables under investigation. Separate models were run for each country (England, Scotland, Wales, and Northern Ireland) and compared to England using interaction terms to identify regional differences in response to the AAN recommendations. Statistical significance was determined using a p-value threshold of 0.05.

To address potential autocorrelation in the data, lag variables were included for each medication. Serial correlation was assessed using the Durbin-Watson statistic, with values between 1.5 and 2.5 considered acceptable. When the statistic fell outside this range, higher-order lag terms were applied to correct for autocorrelation. Specifically, first-order lags were used when residuals were correlated with the previous month, and second-order lags were applied when correlation was observed with values from two months prior [22].

Additionally, the same methods were applied to a separate model that allowed for a six-month lag after the AAN recommendations, to evaluate whether a delayed effect in prescribing patterns was present. This sensitivity analysis, using the lagged model, provided further insights into the potential longer-term impact of the guidelines on prescribing behavior.

This approach allowed for a robust evaluation of prescribing patterns, with appropriate adjustments for multicollinearity, autocorrelation, delayed effects, and interactions between PD medications, providing a clearer understanding of the influence of clinical guidelines and regional differences in PD medication prescriptions.

## Results

**Fig 1:**
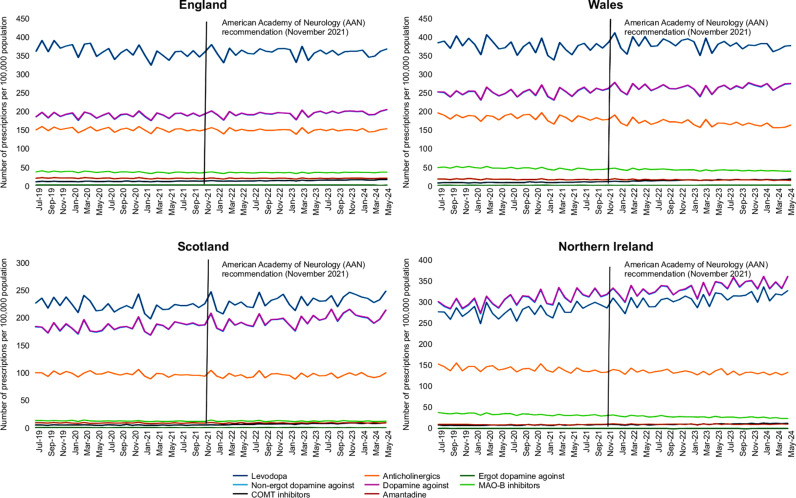
Impact of American Academy of Neurology (AAN) recommendations on Parkinson’s medication prescriptions in England, Wales, Scotland, and Northern Ireland (July 2019–May 2024). This figure illustrates monthly prescribing trends of Parkinson’s disease (PD) medications across the four UK countries. A vertical bold line marks the publication of the AAN guidelines (November 2021). Post-guideline changes are visually evident with Northern Ireland displaying distinct trends in dopamine agonist use.

The study’s key findings showed that levodopa was the most frequently prescribed medication in most regions, followed by DAs, MAO-B inhibitors, COMT inhibitors, and anticholinergics. Regionally, Northern Ireland had the highest overall prescription rates, with Wales, England, and Scotland following in that order (Fig 1, Table 2).

**Table 2:** Comparison of average PD medication prescriptions per 100,000 population across England, Scotland, Wales, and Northern Ireland before and after the AAN recommendation, including 95% confidence intervals.

	England		Scotland		Wales		Northern Ireland	
PD medication	Average number of prescriptions per 100,000 population before the AAN recommendation, including 95% confidence interval (CI)	Average number of prescriptions per 100,000 population after the AAN recommendation, including 95% confidence interval (CI)	Average number of prescriptions per 100,000 population before the AAN recommendation, including 95% confidence interval (CI)	Average number of prescriptions per 100,000 population after the AAN recommendation, including 95% confidence interval (CI)	Average number of prescriptions per 100,000 population before the AAN recommendation, including 95% confidence interval (CI)	Average number of prescriptions per 100,000 population after the AAN recommendation, including 95% confidence interval (CI)	Average number of prescriptions per 100,000 population before the AAN recommendation, including 95% confidence interval (CI)	Average number of prescriptions per 100,000 population after the AAN recommendation, including 95% confidence interval (CI)
Levodopa	360.5 (354.11-366.88)	358.16 (353.99-362.33)	221.8 (217.74-225.86)	230.48 (226.48-234.49)	375.49 (368.84-382.13)	380.37 (375.13-385.62)	279.64 (274.02-285.26)	306.92 (301.61-312.23)
Cabergoline	0.84 (0.81-0.86)	0.73 (0.71-0.75)	0.74 (0.7-0.78)	0.67 (0.63-0.7)	0.94 (0.89-0.99)	1.01 (0.92-1.1)	1.43 (1.37-1.5)	1.31 (1.25-1.37)
Pergolide	0 (0-0)	0 (0-0)	0 (0-0)	0 (0-0)	0 (0-0)	0 (0-0)	0.03 (-0.01-0.07)	0 (0-0)
(All ergots DAs)	0.84 (0.81-0.86)	0.73 (0.71-0.75)	0.74 (0.7-0.78)	0.67 (0.63-0.7)	0.94 (0.89-0.99)	1.01 (0.92-1.1)	1.46 (1.38-1.54)	1.31 (1.25-1.37)
Apomorphine	1.56 (1.51-1.61)	1.44 (1.41-1.46)	1.85 (1.76-1.94)	1.41 (1.36-1.46)	3.3 (3.14-3.46)	2.71 (2.62-2.79)	2.5 (2.38-2.63)	2.14 (2.04-2.24)
Pramipexole	71.65 (70.59-72.71)	77.85 (76.38-79.32)	72.21 (70.89-73.53)	81.41 (79.41-83.41)	92.48 (90.65-94.31)	105.36 (102.91-107.81)	184.62 (180.36-188.87)	211.66 (207.24-216.07)
Ropinirole	95.96 (94.58-97.34)	95.78 (94.63-96.92)	95.57 (93.95-97.19)	99.75 (97.84-101.65)	129.39 (127.28-131.5)	130.06 (127.97-132.14)	92.75 (90.92-94.57)	94.45 (93.21-95.7)
Rotigotine	19.12 (18.77-19.48)	18.44 (18.2-18.68)	12.99 (12.72-13.26)	13.01 (12.76-13.26)	25.38 (24.91-25.85)	25.73 (25.32-26.13)	25.35 (24.69-26)	24.9 (24.4-25.41)
(All Non ergots DAs)	188.29 (185.6-190.98)	193.51 (191.08-195.94)	182.62 (179.54-185.71)	195.57 (191.64-199.51)	250.55 (246.56-254.53)	263.85 (260.4-267.3)	305.21 (299.01-311.42)	333.15 (327.52-338.79)
Rasagiline	29.23 (28.64-29.81)	28.14 (27.77-28.5)	8.29 (8.15-8.42)	10.01 (9.6-10.43)	42.43 (41.4-43.46)	38.84 (38.16-39.52)	31.8 (30.73-32.88)	23.83 (22.99-24.67)
Safinamide	2.55 (2.42-2.69)	4.06 (3.8-4.31)	0.03 (0.01-0.04)	0.04 (0.03-0.05)	0.6 (0.52-0.68)	1.03 (0.95-1.11)	1.68 (1.44-1.92)	4.19 (3.93-4.45)
Selegiline	3.93 (3.73-4.13)	2.26 (1.93-2.58)	4.36 (4.16-4.56)	2.5 (2.09-2.9)	3.76 (3.6-3.92)	2.21 (1.85-2.57)	1.27 (1.21-1.33)	0.85 (0.7-1.01)
(All MAO-B inhibitors)	35.72 (35.02-36.41)	34.45 (34.05-34.85)	12.68 (12.4-12.95)	12.55 (12.28-12.81)	46.79 (45.67-47.91)	42.09 (41.22-42.96)	34.75 (33.83-35.67)	28.87 (28.09-29.66)
Entacapone	28.39 (27.49-29.29)	23.44 (22.96-23.93)	20.19 (19.53-20.85)	17.37 (16.91-17.82)	26.81 (25.73-27.88)	19.63 (18.76-20.5)	21.91 (21.52-22.3)	20.88 (20.44-21.31)
Opicapone	3.84 (3.54-4.15)	6.94 (6.51-7.37)	0.39 (0.32-0.45)	2.93 (2.37-3.49)	5.03 (4.63-5.43)	10.97 (10.28-11.66)	1.46 (1.26-1.65)	3.79 (3.31-4.26)
Tolcapone	0.06 (0.05-0.07)	0.03 (0.02-0.03)	0.05 (0.04-0.06)	0.03 (0.02-0.04)	0.05 (0.02-0.09)	0 (0-0)	0.31 (0.27-0.34)	0.16 (0.14-0.18)
(All COMT inhibitors)	10.69 (10.47-10.91)	13.11 (12.65-13.57)	5.36 (5.2-5.52)	7.43 (7.03-7.83)	8.63 (8.26-8.99)	14.34 (13.68-15.01)	9.34 (9.02-9.65)	11.65 (11.18-12.12)
Benztropine	0.03 (0.02-0.04)	0.02 (0.01-0.02)	0 (0-0)	0 (0-0)	0.05 (0.03-0.06)	0.01 (0-0.02)	0 (0-0)	0 (0-0)
Orphenadrine	0.91 (0.89-0.94)	0.75 (0.72-0.77)	0.85 (0.82-0.87)	0.67 (0.63-0.7)	1.27 (1.2-1.33)	0.94 (0.88-1)	3.25 (3.09-3.4)	2.51 (2.4-2.63)
Procyclidine	131.7 (129.94-133.46)	129.69 (128.29-131.1)	85.58 (84.27-86.89)	83.66 (82.37-84.96)	162.06 (159.47-164.64)	148.92 (146.21-151.63)	124.46 (122.09-126.83)	116.9 (115.39-118.42)
Trihexyphenidyl	17.88 (17.63-18.13)	18.2 (17.98-18.41)	11.45 (11.24-11.66)	11.41 (11.15-11.68)	20.76 (20.33-21.2)	18.45 (18.06-18.84)	14.74 (14.43-15.06)	15.33 (15.02-15.64)
(All anticholinergics)	150.53 (148.51-152.54)	148.65 (147.05-150.25)	97.88 (96.4-99.36)	95.74 (94.24-97.24)	184.13 (181.13-187.14)	168.32 (165.24-171.4)	142.45 (139.86-145.04)	134.75 (133.04-136.46)

Focusing on Levodopa, prescription rates in England remained relatively stable, decreasing slightly from 360.5 to 358.16 prescriptions per 100,000 population after the specified period. Northern Ireland saw an increase from 279.64 to 306.92, while Wales experienced a small rise from 375.49 to 380.37, and Scotland showed a more moderate increase from 221.8 to 230.48.

**Fig 2:**
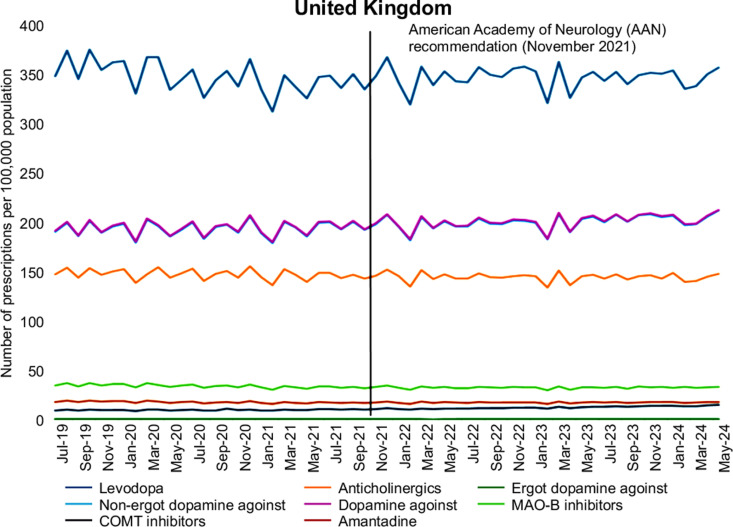
Influence of the American Academy of Neurology (AAN) recommendations on Parkinson’s disease medication prescribing trends across the United Kingdom (July 2019–May 2024). This figure presents aggregated monthly data on Parkinson’s disease (PD) prescriptions across the UK. The vertical bold line marks the point of AAN guideline publication, distinguishing the pre- and post-guideline periods.

For DAs, Northern Ireland exhibited a unique trend where DAs were prescribed more frequently than Levodopa, with DAs increasing from 305.21 to 333.15 per 100,000 population, surpassing Levodopa prescriptions, which rose to 306.92. This distinct behavior, possibly influenced by factors such as a younger population in Northern Ireland, will be explored in more detail in the Discussion section. In contrast, Levodopa remained the most prescribed medication in the other regions. England showed a smaller rise in DAs, from 188.29 to 193.51, while Scotland and Wales saw increases from 182.62 to 195.57 and from 250.55 to 263.85, respectively. These trends are summarized for the entire UK in Fig 2, illustrating the overall prescribing patterns for Levodopa and DAs and other PD medications.

The segmented regression analysis for Levodopa across the four UK countries (Table 3) highlighted distinct trends and responses to the AAN recommendations. In England, Levodopa prescribing exhibited a significant decreasing trend (-0.105, p = 0.044), followed by a marked increase after the AAN policy implementation (6.79, p < 0.001), reflecting an immediate impact of the guidelines. This represents a substantial shift in prescribing behavior, particularly considering the prior stable or declining trends. However, in the six-month lag model, the AAN recommendation had no significant effect on Levodopa prescribing, leading to its exclusion from the backward logistic regression model (Table 4). In Scotland, Wales, and Northern Ireland (NI), there was no significant impact of the AAN recommendation on Levodopa prescribing, and the variable was excluded from the analysis. Wales showed a slight increase in impact compared to England (0.035, p < 0.001) (Table 3).

**Table 3:** Segmented regression analysis of PD medication trends examining the impact of AAN recommendation.

Prescribing Factors PD medications	Country	Complete trend analysis (July 2019 - May 2024)^1^	Impact of the American Academy of Neurology (AAN) recommendation in November 2021 (binary variable)	Effect of country compared to the reference (England), following the interaction term that multiplies the AAN effect with the country	Levodopa prescribing impact	All DAs prescribing impact	MAO-B inhibitors prescribing impact	COMT inhibitors prescribing impact	Amantadine prescribing impact	Anticholinergics prescribing impact	Adjusted R^2^	Durbin- Watson statistics
Levodopa	**England**	**-0.105 (-0.207--0.003) (0.044)**	**6.79 (3.556-10.024) (<.001)**	NA	NA	Removed (VIF^2^ = 11.2)	Excluded^3^	Excluded	**11.92 (9.78-14.061) (<.001)**	**0.908 (0.544-1.272) (<.001)**	0.951	1.521 (after Second-order lag)
	**Scotland**	Removed (VIF = 12.09)	Excluded	Excluded	NA	0.285 (0.071-0.498) (0.01)	2.944 (0.779-5.11) (0.009)	2.96 (1.465-4.454) (<.001)	3.963 (0.797-7.13) (0.015)	0.728 (0.317-1.14) (<.001)	0.893	1.80 (after First-order lag)
	**Wales**	Removed (VIF = 44.51)	Excluded	0.035 (0.016-0.054) (<.001)	NA	0.634 (0.426-0.842) (<.001)	Removed (VIF = 14.30)	Removed (VIF = 23.20)	5.121 (2.823-7.419) (<.001)	0.627 (0.295-0.959) (<.001)	0.873	1.61 (after Second-order lag)
	**NI** ^**^	**Removed (VIF = 54.10)**	Excluded	Excluded	NA	0.654 (0.498-0.81) (<.001)	Excluded	2.488 (0.586-4.391) (0.011)	3.454 (1.046-5.861) (0.006)	Excluded	0.992	2.31 (after First-order lag)
All DAs	**England**	**0.269 (0.233-0.305) (<.001)**	Excluded	NA	Removed (VIF = 15.04)	NA	Removed (VIF = 12.52)	Removed (VIF = 11.41)	Removed (VIF = 14.40)	1.307 (1.182-1.432) (<.001)	0.915	1.87 (after First-order lag)
	**Scotland**	0.537 (0.405-0.669) (<.001)	**-3.904 (-7.573--0.235) (0.037)**	Removed (VIF = 74.70)	0.241 (0.034-0.448) (0.024)	NA	3.235 (0.996-5.473) (0.005)	Excluded	3.867 (1.073-6.661) (0.008)	0.513 (0.096-0.93) (0.017)	0.921	1.57 (after First-order lag)
	**Wales**	0.767 (0.617-0.916) (<.001)	Excluded	Excluded	0.313 (0.197-0.428) (<.001)	NA	Excluded	Excluded	1.746 (0.078-3.414) (0.041)	0.515 (0.311-0.718) (<.001)	0.931	1.71 (after Third-order lag)
	**NI**	1.002 (0.619-1.385) (<.001)	**-5.668 (-11.371-0.034) (0.051)**	Excluded	0.532 (0.332-0.732) (<.001)	NA	Removed (VIF = 21.90)	Excluded	Excluded	0.942 (0.475-1.408) (<.001)	0.933	1.67 (after First-order lag)
MAO-B inhibitors^**^	**England**	Removed (VIF = 34.2)	-1.027 (-1.318--0.737) (<.001)	NA	0.097 (0.087-0.107) (<.001)	Excluded	NA	Removed (VIF-23.6)	Removed (VIF = 14.9)	Excluded	0.881	1.64 (after First-order lag)
	**Scotland**	-0.068 (-0.084--0.053) (<.001)	Excluded	**0.021 (0.009-0.032) (<.001)**	Excluded	0.048 (0.034-0.063) (<.001)	NA	0.251 (0.057-0.445) (0.012)	Excluded	Excluded	0.732	2.28 (after First-order lag)
	**Wales**	-0.197 (-0.223--0.171) (<.001)	Excluded	Excluded	0.098 (0.083-0.113) (<.001)	Excluded	NA	Excluded	Excluded	Excluded	0.939	1.51 (after First-order lag)
	**NI**	-0.284 (-0.304--0.263) (<.001)	Excluded	Excluded	0.061 (0.027-0.095) (<.001)	0.028 (-0.005-0.061) (0.094)	NA	Excluded	Excluded	Excluded	0.954	2.04 (after First-order lag)
COMT inhibitors^**^	**England**	0.079 (0.065-0.092) (<.001)	**-0.311 (-0.607--0.016) (0.039)**	**NA**	Excluded	Excluded	Excluded	NA	0.7 (0.605-0.795) (<.001)	Excluded	0.968	2.1 (after First-order lag)
	**Scotland**	0.034 (0.021-0.046) (<.001)	Excluded	**Removed (VIF = 69.10)**	0.039 (0.029-0.049) (<.001)	Excluded	Excluded	NA	Excluded	Excluded	0.911	2.26 (after First-order lag)
	**Wales**	0.107 (0.076-0.138) (<.001)	Excluded	**Removed (VIF = 14.90)**	0.035 (0.026-0.044) (<.001)	Excluded	Excluded	NA	Excluded	Excluded	0.974	2.23 (after First-order lag)
	**NI**	0.066 (0.046-0.086) (<.001)	**-0.562 (-1.127-0.003) (0.051)**	**Removed (VIF = 23.10)**	0.036 (0.024-0.048) (<.001)	Excluded	Excluded	NA	Excluded	Excluded	0.885	1.85 (after First-order lag)
Amantadine	**England**	Excluded	Excluded	NA	0.035 (0.028-0.041) (<.001)	Excluded	0.218 (0.158-0.279) (<.001)	Removed (VIF = 21.4)	NA	Excluded	0.958	1.79 (after First-order lag)
	**Scotland**	-0.014 (-0.021--0.007) (<.001)	Excluded	Excluded	0.025 (0.009-0.042) (0.003)	0.025 (0.005-0.045) (0.017)	Excluded	Excluded	NA	Excluded	0.746	2.16 (after First-order lag)
	**Wales**	-0.062 (-0.079--0.046) (<.001)	Excluded	Excluded	0.026 (0.005-0.047) (0.018)	0.035 (0-0.07) (0.048)	Excluded	Excluded	NA	Excluded	0.821	1.99 (after First-order lag)
	**NI**	-0.03 (-0.048--0.012) (0.001)	Excluded	0.076 (0.045-0.106) (<.001)	0.026 (0.015-0.037) (<.001)	Excluded	Excluded	Excluded	NA	Excluded	0.630	2.12 (after First-order lag)
Anticholinergics	**England**	0.079 (0.065-0.092) (<.001)	-0.311 (-0.607--0.016) (0.039)	**NA**	Excluded	Excluded	Excluded	Excluded	0.7 (0.605-0.795) (<.001)	NA	0.968	2.18 (after First-order lag)
	**Scotland**	-0.203 (-0.253--0.154) (<.001)	Excluded	**Removed (VIF-83.2)**	0.156 (0.045-0.267) (0.007)	0.207 (0.071-0.344) (0.004)	Excluded	Excluded	Excluded	NA	0.753	1.99 (after First-order lag)
	**Wales**	-0.637 (-0.752--0.521) (<.001)	-5.529 (-9.259--1.8) (0.004)	Excluded	Excluded	0.634 (0.537-0.731) (<.001)	Excluded	Excluded	Excluded	NA	0.899	1.56 (after Second-order lag)
	**NI**	-0.592 (-0.666--0.518) (<.001)	Excluded	Excluded	0.114 (-0.009-0.238) (0.069)	0.232 (0.113-0.351) (<.001)	Excluded	Excluded	Excluded	NA	0.820	2.18 (after First-order lag)

**Notes:** 1. Coefficients from regression analysis are presented alongside their 95% confidence intervals and p value.

2. NI stands for Northern Ireland, DAs for Dopamine Agonists, MAO-B inhibitors for Monoamine Oxidase-B inhibitors, COMT inhibitors for Catechol-O-methyltransferase inhibitors, and VIF for Variance Inflation Factor.

3. Excluded from the final model in the backward linear regression due to lack of significant contribution to the dependent variable

**Table 4:** Segmented regression analysis of PD medication trends with a 6-month lag to assess the impact of AAN recommendation.

Prescribing Factors PD medications	Country	Complete trend analysis (July 2019 - May 2024)*	Impact of the American Academy of Neurology (AAN) recommendation in November 2021 (binary variable) with a 6-Month Lag	Effect of country compared to the reference (England), following the interaction term that multiplies the AAN effect with the country	Levodopa prescribing impact	All DAs prescribing impact	MAO-B inhibitors prescribing impact	COMT inhibitors prescribing impact	Amantadine prescribing impact	Anticholinergics prescribing impact	Adjusted R^2^	Durbin- Watson statistics
Levodopa	**England**	^Removed (VIF** = 14.21)^	**Excluded** ^***^	NA	NA	0.888 (0.53-1.245) (<.001)	**Excluded**	**-1.611 (-2.692--0.53) (0.004)**	**11.307 (9.031-13.582) (<.001)**	**Excluded**	0.946	1.52 (after First-order lag)
	**Scotland**	**Excluded**	**Excluded**	**Excluded**	NA	0.285 (0.071-0.498) (0.01)	2.944 (0.779-5.11) (0.009)	2.96 (1.465-4.454) (<.001)	3.963 (0.797-7.13) (0.015)	0.728 (0.317-1.14) (<.001)	0.893	1.80 (after First-order lag)
	**Wales**	^Removed (VIF** = 43.21)^	**Excluded**	**Excluded**	NA	0.664 (0.424-0.903) (<.001)	3.466 (2.456-4.475) (<.001)	2.593 (1.366-3.82) (<.001)	2.68 (0.284-5.075) (0.029)	**Excluded**	0.909	1.75 (after Second-order lag)
	**NI** ^**^	**Excluded**	**Excluded**	**Excluded**	NA	0.654 (0.498-0.81) (<.001)	Removed (VIF = 19.51)	2.488 (0.586-4.391) (0.011)	3.454 (1.046-5.861) (0.006)	**Excluded**	0.922	2.32 (after First-order lag)
All DAs	**England**	**0.33 (0.276-0.384) (<.001)**	**-1.754 (-4.263--0.645) (0.04)**	NA	Removed (VIF = 11.50)	NA	**Excluded**	**Excluded**	4.17 (2.944-5.396) (<.001)	0.708 (0.511-0.906) (<.001)	0.954	1.51 (after First-order lag)
	**Scotland**	0.556 (0.433-0.679) (<.001)	**Excluded**	-0.026 (-0.044--0.008) (0.006)	0.261 (0.059-0.463) (0.012)	NA	3.157 (1.036-5.278) (0.004)	**Excluded**	4.164 (1.474-6.853) (0.003)	0.481 (0.076-0.885) (0.021)	0.926	1.61 (after Second-order lag)
	**Wales**	0.818 (0.693-0.944) (<.001)	**-4.591 (-7.829--1.353) (0.006)**	**Excluded**	0.362 (0.258-0.465) (<.001)	NA	**Excluded**	**Excluded**	1.276 (-0.236-2.789) (0.096)	0.438 (0.259-0.617) (<.001)	0.939	1.58 (after Second-order lag)
	**NI**	^**Removed (VIF = 19.50)**^	**Excluded**	**Excluded**	0.883 (0.763-1.003) (<.001)	NA	-1.435 (-2.498--0.372) (0.009)	**Excluded**	**Excluded**	0.562 (0.076-1.049) (0.024)	0.912	1.71 (after Second-order lag)
MAO-B inhibitors^**^	**England**	-0.019 (-0.027--0.011) (<.001)	**Excluded**	NA	**Excluded**	**Excluded**	NA	Removed (VIF = 23.04)	1.694 (1.539-1.848) (<.001)	**Excluded**	0.917	1.81 (after First-order lag)
	**Scotland**	-0.054 (-0.067--0.041) (<.001)	**Excluded**	**0.02 (0.008-0.031) (0.001)**	**Excluded**	0.061 (0.049-0.073) (<.001)	NA	**Excluded**	**Excluded**	**Excluded**	0.698	2.11 (after First-order lag)
	**Wales**	-0.197 (-0.223--0.171) (<.001)	**Excluded**	**Excluded**	0.098 (0.083-0.113) (<.001)	**Excluded**	NA	**Excluded**	**Excluded**	**Excluded**	0.939	1.51 (after First-order lag)
	**NI**	-0.311 (-0.345--0.278) (<.001)	**Excluded**	**Excluded**	0.081 (0.063-0.098) (<.001)	**Excluded**	NA	**Excluded**	**Excluded**	**Excluded**	0.956	1.61 (after First-order lag)
COMT inhibitors^**^	**England**	0.07 (0.059-0.081) (<.001)	**Excluded**	**NA**	**Excluded**	**Excluded**	Removed (VIF = 12.70)	NA	0.688 (0.591-0.786) (<.001)	**Excluded**	0.966	1.97 (after First-order lag)
	**Scotland**	0.022 (0.01-0.035) (<.001)	**Excluded**	**0.073 (0.035-0.112) (<.001)**	0.035 (0.026-0.044) (<.001)	**Excluded**	**Excluded**	NA	**Excluded**	**Excluded**	0.929	2.21 (after First-order lag)
	**Wales**	0.112 (0.085-0.138) (<.001)	**Excluded**	**0.112 (0.063-0.16) (<.001)**	0.031 (0.023-0.038) (<.001)	**Excluded**	Removed (VIF = 16.30)	NA	**Excluded**	**Excluded**	0.980	2.21 (after First-order lag)
	**NI**	0.051 (0.037-0.066) (<.001)	**Excluded**	**Excluded**	0.036 (0.024-0.048) (<.001)	**Excluded**	**Excluded**	NA	**Excluded**	**Excluded**	0.879	1.69 (after First-order lag)
Amantadine	**England**	Removed (VIF = 23.62)	**Excluded**	NA	0.035 (0.028-0.041) (<.001)	**Excluded**	0.218 (0.158-0.279) (<.001)	**Excluded**	NA	**Excluded**	0.958	1.79 (after First-order lag)
	**Scotland**	-0.014 (-0.021--0.007) (<.001)	Removed (VIF = 15.95)	**Excluded**	0.025 (0.009-0.042) (0.003)	0.025 (0.005-0.045) (0.017)	**Excluded**	**Excluded**	NA	**Excluded**	0.746	2.16 (after First-order lag)
	**Wales**	-0.062 (-0.079--0.046) (<.001)	**Excluded**	**Excluded**	0.026 (0.005-0.047) (0.018)	0.035 (0-0.07) (0.048)	**Excluded**	**Excluded**	NA	**Excluded**	0.811	1.99 (after First-order lag)
	**NI**	**Excluded**	**Excluded**	**Excluded**	0.028 (0.021-0.036) (<.001)	**Excluded**	**Excluded**	**Excluded**	NA	**Excluded**	0.481	2.06 (after First-order lag)
Anticholinergics	**England**	-0.182 (-0.207--0.156) (<.001)	**Excluded**	**NA**	**Excluded**	0.672 (0.611-0.732) (<.001)	**Excluded**	**Excluded**	**Excluded**	NA	0.903	2.28 (after First-order lag)
	**Scotland**	-0.203 (-0.253--0.154) (<.001)	**Excluded**	**Excluded**	0.156 (0.045-0.267) (0.007)	0.207 (0.071-0.344) (0.004)	**Excluded**	**Excluded**	**Excluded**	NA	0.753	1.99 (after First-order lag)
	**Wales**	-0.765 (-0.837--0.692) (<.001)	**Excluded**	**Excluded**	**Excluded**	0.611 (0.508-0.713) (<.001)	Removed (VIF = 18. 50)	**Excluded**	**Excluded**	NA	0.886	1.87 (after First-order lag)
	**NI**	-0.592 (-0.666--0.518) (<.001)	**Excluded**	**Excluded**	0.114 (-0.009-0.238) (0.069)	0.232 (0.113-0.351) (<.001)		**Excluded**	**Excluded**	NA	0.820	2.18 (after First-order lag)

**Notes:** 1. Coefficients from regression analysis are presented alongside their 95% confidence intervals and p value.

2. NI stands for Northern Ireland, DAs for Dopamine Agonists, MAO-B inhibitors for Monoamine Oxidase-B inhibitors, COMT inhibitors for Catechol-O-methyltransferase inhibitors, and VIF for Variance Inflation Factor.

3. Excluded from the final model in the backward linear regression due to lack of significant contribution to the dependent variable.

For DAs, England demonstrated a consistent increase in prescribing (0.269, p < 0.001) with no significant immediate effect from the AAN recommendations. Although the immediate effect was not significant, the consistent increase in prescribing suggests ongoing adoption of DAs as a first-line treatment. However, in the six-month lag model (Table 4), a significant reduction in prescribing rates was observed (-1.754, p = 0.04). Both Scotland and Wales followed similar patterns, showing positive trends pre-AAN and notable decreases post-AAN (-3.904, p = 0.037 in Scotland in the original model, and -4.591, p = 0.006 in Wales in the six-month lag model) (Tables 3 and 4). The only significant country effect was in the six-month lag model, where Scotland was less impacted by the AAN recommendations in reducing DA prescriptions compared to England (-0.026, p = 0.006).

MAO-B inhibitors showed significant decreases across most regions in both the original and six-month lag models (Tables 3 and 4). England exhibited the largest reduction after the AAN recommendations (-1.027, p < 0.001). In terms of the country effect, Scotland was more impacted by the AAN recommendations compared to England in both the original (0.021, p < 0.001) and six-month lag models (0.02, p = 0.001) (Tables 3 and 4).

COMT inhibitors showed significant increases in prescribing across all countries (Table 3), with England displaying a notable rise (0.079, p < 0.001). However, post-AAN, prescribing decreased in England (-0.311, p = 0.039) in the original model, though no significant effect was found in the six-month lag model.

The amantadine and anticholinergic models, shown in Tables 3 and 4, indicate a significant reduction in anticholinergic prescribing following the AAN recommendation in both England and Wales. The decline was more pronounced in Wales (-5.529, p = 0.004) compared to England (-0.311, p = 0.039) in the original model. However, in the six-month lag model, this reduction was not found to be significant.

In terms of model quality, the adjusted R² values across the models ranged from 0.630 to 0.992, indicating strong explanatory power for most models (Tables 3 and 4). The Durbin-Watson statistics were within the acceptable range of 1.5 to 2.5 for all models, confirming that autocorrelation in the residuals was effectively addressed, with first- or second-order lags applied where necessary to improve model accuracy.

## Discussion

This study aimed to examine PD medication prescribing patterns across the UK and assess the impact of the AAN and NICE guidelines. It focused on how Levodopa and DAs were prescribed between July 2019 and May 2024. The results revealed that levodopa was the most prescribed medication across all regions, with a significant increase in England following the AAN guideline introduction. In contrast, other regions showed more stable trends, and Northern Ireland exhibited a distinct pattern, with DAs prescribed more often than levodopa. Additionally, Scotland and Wales were less responsive to the AAN guidelines. These findings highlight how clinical guidelines influence PD prescribing practices, with regional variations suggesting possible demographic or healthcare system factors. The AAN guidelines, published in November 2021, recommend starting Levodopa earlier in the disease for its superior motor symptom relief, particularly in patients over 70. Despite the risk of long-term complications like dyskinesia, the AAN emphasizes Levodopa’s immediate motor benefits [14]. In contrast, the NICE guidelines from 2017 prioritize DAs for younger patients to delay Levodopa’s motor complications. While both guidelines acknowledge the efficacy of Levodopa, AAN supports its early use, whereas NICE recommends delaying Levodopa and using DAs in younger patients to reduce long-term risks [11].

Findings from this study indicate that Levodopa remained the most prescribed PD medication across all UK regions, with England experiencing an initial decline in Levodopa use, followed by a significant increase after the AAN guidelines were introduced. However, the six-month lag model suggests that the AAN recommendations did not have a lasting effect, as prescribing trends eventually stabilized and may reflect a renewed consideration of DAs for early-stage PD in England. Scotland, Wales, and Northern Ireland, where Levodopa consistently remained more commonly prescribed than DAs, exhibited stable trends with no notable changes after the AAN guidelines, suggesting closer adherence to the NICE guidelines in these regions. Northern Ireland stands out, having a higher rate of DA prescriptions than Levodopa compared to the other regions.

The higher Levodopa prescribing rates across the UK align with similar findings in countries like the USA, Japan, and Taiwan [23–27]. This suggests that, despite different healthcare systems, there is an international convergence on levodopa as the dominant first-line treatment, particularly as newer evidence and guidelines emerge. The trends in most regions of the UK suggest that Levodopa use has stabilized. The exception is Northern Ireland, where DAs were prescribed at higher rates than Levodopa, with 320.5 DA prescriptions per 100,000 population compared to 293.12 for Levodopa. While it is difficult to pinpoint the exact reasons for this trend, one possible explanation is Northern Ireland’s younger population. According to 2021 estimates, Northern Ireland has the lowest proportion of individuals aged 65+ (17.93%) and 85+ (2.17%) in the UK, while it has the highest proportion of those aged 0–15 (20.45%) [20]. This demographic shift could influence prescribing patterns, as younger patients may be more likely to be started on DAs in line with the NICE guidelines. Another explanation could be regional variations in prescriber behavior, though further investigation, particularly with patient-level data, is needed to explore this hypothesis.

The differences in prescribing patterns across the UK may also reflect variations in healthcare infrastructure and access to specialist care. In regions like England, where neurologists and Parkinson’s specialists are more widely available, AAN guidelines promoting earlier use of Levodopa might be more readily implemented. On the other hand, in Northern Ireland, where only 3% of neurological admissions are under neurology care compared to 11% in England [28], general practitioners often manage PD, leading to greater adherence to NICE guidelines, which favor DAs for younger patients. These regional differences underscore the importance of considering healthcare systems and physician expertise when evaluating the implementation of clinical guidelines. Understanding these disparities can guide future updates to reflect local healthcare realities.

Economic factors likely contribute to the regional differences in PD prescribing patterns observed in this study. Levodopa’s proven cost-effectiveness compared to DAs plays a critical role in resource-constrained healthcare systems like the NHS [29,30]. Levodopa’s effectiveness in providing immediate symptom relief and its long-term economic advantage, especially when considering the management of side effects, may explain its widespread use in most regions. In contrast, Northern Ireland, which may have fewer healthcare resources, shows a higher preference for DAs, potentially reflecting a short-term focus on managing motor symptoms and delaying costly complications. This approach, although appearing to reduce immediate burdens, overlooks Levodopa’s long-term cost benefits. Additionally, the lack of strategic resource allocation in Northern Ireland—where resources are distributed on a pro rata basis without clear strategic direction—contributes to inefficiencies and low productivity compared to England, further complicating the long-term management of PD [31]. The stable DA prescribing in Scotland and Wales may also reflect a cautious strategy. These findings underscore the need for considering both economic and patient-centered factors when developing treatment guidelines, particularly in regions with limited resources.

The impact of clinical guideline adherence on patients’ long-term quality of life deserves greater attention in future research and health policy. While the clinical efficacy and cost-effectiveness of levodopa are well established, its potential to improve daily functioning, autonomy, and psychological well-being, especially among older adults, adds further weight to its use as a first-line therapy [10]. Differences in regional prescribing patterns, as observed in this study, may lead to variable patient experiences and long-term outcomes. [10]. For instance, delayed initiation of levodopa in favor of dopamine agonists might postpone motor symptom relief, affecting patients’ independence and social participation during critical stages of disease progression [32]. Conversely, early levodopa use may offer improved quality of life despite potential risks such as dyskinesia, which many patients consider a manageable trade-off. Understanding how such trade-offs are perceived by patients in different regions, and how they relate to prescribing practices, can guide the development of more individualized and patient-centered treatment approaches [32]. Ultimately, integrating quality of life metrics into prescribing evaluations may help ensure that clinical decisions align not only with pharmacoeconomic goals, but also with what matters most to patients themselves [32].

This study’s strength lies in its comprehensive use of GP records, capturing the majority of PD prescriptions in the UK. This provides a robust dataset for examining regional variations in prescribing trends. Furthermore, this is the first study to compare PD medication use across all four UK countries, building on earlier research while utilizing segmented regression with conservative quality measures like variance inflation factor (VIF) and Durbin-Watson tests, ensuring the results are both valid and reliable.

However, there are certain limitations to consider. One notable shortcoming is the reliance on publicly available prescription data, which lacks patient-level granularity. The absence of detailed patient demographics, clinical histories, or disease stages means that prescribing patterns cannot be fully contextualized in relation to individual patient characteristics. For instance, data on age, disease progression, and co-morbidities would be essential for understanding whether younger patients are being prescribed DAs in accordance with NICE guidelines or if other factors are influencing treatment decisions. The lack of such data limits the ability to fully interpret regional prescribing variations. Additionally, the interrupted time series (ITS) approach, while effective in identifying immediate changes at the intervention point, may not fully capture subtle shifts in prescribing trends over time. This modeling limitation may influence how gradual changes in prescribing behavior are detected. Furthermore, regional differences in prescribing practices could reflect unmeasured socioeconomic or healthcare infrastructure factors. The variations observed across the UK regions, particularly in Northern Ireland, could be influenced by differences in access to care, socioeconomic status, or healthcare policies, which were not accounted for in this analysis. Lastly, the lack of diagnostic confirmation means that some prescriptions, especially for anticholinergics, may have been issued for conditions other than PD, such as drug-induced parkinsonism or dystonia. However, medications like Levodopa and DAs are predominantly used for PD, which reinforces the reliability of the findings for these drugs.

## Conclusion

In conclusion, this study provides valuable insights into how clinical guidelines influence PD medication prescribing across the UK, underscoring the varying impacts of the AAN and NICE guidelines. While Levodopa remains the most commonly prescribed PD treatment overall, trends suggest that in England, there may be a gradual shift back toward DAs for early PD management, in line with NICE recommendations. Meanwhile, other UK countries, including Scotland, Wales, and Northern Ireland, demonstrate more stable prescribing patterns, possibly reflecting continued adherence to the NICE guidelines. The study’s strength lies in its use of comprehensive prescribing data and robust statistical methods, but the absence of patient-level data and diagnostic confirmation presents limitations. Despite these constraints, the findings provide a solid foundation for future research. Key areas for investigation could include exploring patient-level data to validate the trends observed at the regional level and examining the long-term health outcomes associated with different prescribing practices. Additionally, expanding research to include cost-effectiveness analyses or studies on healthcare resource allocation could offer valuable insights into the economic implications of these trends. Future studies could also focus on understanding the factors driving regional adherence or divergence from guidelines, such as patient demographics, GP attitudes, and access to healthcare, which could provide actionable insights for optimizing prescribing practices.

## References

[1] Bloem BR, Okun MS, Klein C. Parkinson’s disease. Lancet. 2021;397(10291):2284–303. doi: 10.1016/S0140-6736(21)00218-X 33848468

[2] Lee A, Gilbert RM. Epidemiology of Parkinson disease. Neurol Clin. 2016;34(4):955–65. doi: 10.1016/j.ncl.2016.06.012 27720003

[3] Okunoye O, Marston L, Walters K, Schrag A. Change in the incidence of Parkinson’s disease in a large UK primary care database. NPJ Parkinsons Dis. 2022;8(1):23. doi: 10.1038/s41531-022-00284-0 35292689 PMC8924194

[4] Lix LM, Hobson DE, Azimaee M, Leslie WD, Burchill C, Hobson S. Socioeconomic variations in the prevalence and incidence of Parkinson’s disease: a population-based analysis. J Epidemiol Community Health. 2010;64(4):335–40. doi: 10.1136/jech.2008.084954 19679711

[5] Rocca WA, Bower JH, McDonnell SK, Peterson BJ, Maraganore DM. Time trends in the incidence of parkinsonism in Olmsted county, Minnesota. Neurology. 2001;57(3):462–7. doi: 10.1212/wnl.57.3.462 11502914

[6] Varden R, Walker R, O’Callaghan A. No trend to rising rates: a review of Parkinson’s prevalence studies in the United Kingdom. Parkinsonism Relat Disord. 2024;128:107015. doi: 10.1016/j.parkreldis.2024.107015 38876845

[7] Suchowersky O, Gronseth G, Perlmutter J, Reich S, Zesiewicz T, Weiner WJ, et al. Practice parameter: neuroprotective strategies and alternative therapies for Parkinson disease (an evidence-based review): report of the quality standards subcommittee of the American academy of neurology. Neurology. 2006;66(7):976–82. doi: 10.1212/01.wnl.0000206363.57955.1b 16606908

[8] Zhang J, Tan LC-S. Revisiting the medical management of Parkinson’s disease: levodopa versus dopamine agonist. Curr Neuropharmacol. 2016;14(4):356–63. doi: 10.2174/1570159x14666151208114634 26644151 PMC4876591

[9] Inzelberg R, Nisipeanu P, Schechtman E. Practice parameter: initiation of treatment for Parkinson’s disease: an evidence-based review. Neurology. 2002;59(8):1292; author reply 1292. doi: 10.1212/wnl.59.8.1292 12391377

[10] Gray R, Ives N, Rick C, Patel S, Gray A, PD Med Collaborative Group, et al. Long-term effectiveness of dopamine agonists and monoamine oxidase B inhibitors compared with levodopa as initial treatment for Parkinson’s disease (PD MED): a large, open-label, pragmatic randomised trial. Lancet. 2014;384(9949):1196–205. doi: 10.1016/S0140-6736(14)60683-8 24928805

[11] Parkinson’s disease in adults: diagnosis and management. National Institute for Health and Care Excellence; 2017. 28787113

[12] Kalilani L, Friesen D, Boudiaf N, Asgharnejad M. The characteristics and treatment patterns of patients with Parkinson’s disease in the United States and United Kingdom: a retrospective cohort study. PLoS One. 2019;14(11):e0225723. doi: 10.1371/journal.pone.0225723 31756215 PMC6874315

[13] Orayj K, Akbari A, Lacey A, Smith M, Pickrell O, Lane EL. Factors affecting the choice of first-line therapy in Parkinson’s disease patients in Wales: a population-based study. Saudi Pharm J. 2021;29(2):206–12. doi: 10.1016/j.jsps.2021.01.004 33679182 PMC7910137

[14] Pringsheim T, Day GS, Smith DB, Rae-Grant A, Licking N, Armstrong MJ, et al. Dopaminergic therapy for motor symptoms in early parkinson disease practice guideline summary: a report of the AAN guideline subcommittee. Neurology. 2021;97(20):942–57. doi: 10.1212/WNL.0000000000012868 34782410 PMC8672433

[15] Bennett institute for applied data science U of O. OpenPrescribing. 2024. Available: https://openprescribing.net/

[16] NHS Wales Shared Services Partnership. General practice prescribing data extract. In: NHS wales shared services partnership [Internet]. 2024 [cited 27 Sep 2024]. Available: https://nwssp.nhs.wales/ourservices/primary-care-services/general-information/data-and-publications/prescribing-data-extracts//.

[17] Public Health Scotland. Monthly prescribing activity data. In: Public health Scotland [Internet]. 2024. [cited 27 Sep 2024]. Available: https://publichealthscotland.scot/publications/monthly-prescribing-activity-data/monthly-prescribing-activity-data-data-for-june-2024//

[18] OpenData NI. GP prescribing data. OpenData NI. 2024. Accessed 2024 September 27 https://admin.opendatani.gov.uk/dataset?_tags_limit=0&license_id=uk-ogl&tags=PrimaryCare&groups=health

[19] Orayj K. Pharmacotherapeutic interventions in Parkinson’s disease: investigating prescribing factors and health outcomes. Cardiff University; 2020.

[20] Office for National Statistics. Population estimates. Office for National Statistics. 2024. Accessed 2024 September 27 Available from: https://www.ons.gov.uk/

[21] O’brien RM. A caution regarding rules of thumb for variance inflation factors. Qual Quant. 2007;41(5):673–90. doi: 10.1007/s11135-006-9018-6

[22] Bhargava A, Franzini L, Narendranathan W. Serial Correlation and the Fixed Effects Model. Rev Econ Stud. 1982;49(4):533. doi: 10.2307/2297285

[23] Nakaoka S, Ishizaki T, Urushihara H, Satoh T, Ikeda S, Yamamoto M, et al. Prescribing pattern of anti-Parkinson drugs in Japan: a trend analysis from 2005 to 2010. PLoS One. 2014;9(6):e99021. doi: 10.1371/journal.pone.0099021 24906013 PMC4048287

[24] Guo Y-J, Liao Y-C, Lin C-H, Chang M-H. Initial medication in patients of newly diagnosed Parkinson’s disease in Taiwan. PLoS One. 2014;9(9):e107465. doi: 10.1371/journal.pone.0107465 25222829 PMC4164642

[25] Swarztrauber K, Koudelka C, Brodsky MA. Initial pharmacotherapy in a population of veterans with Parkinson disease. Neurology. 2006;66(9):1425–6. doi: 10.1212/01.wnl.0000210433.49727.40 16682678

[26] Orayj K, Lane E. Patterns and determinants of prescribing for Parkinson’s Disease: a systematic literature review. Parkinsons Dis. 2019;2019:9237181. doi: 10.1155/2019/9237181 31781365 PMC6875178

[27] Huse DM, Castelli-Haley J, Orsini LS, Lenhart G, Abdalla JA. Patterns of initial pharmacotherapy for Parkinson’s disease in the United States. J Geriatr Psychiatry Neurol. 2006;19(2):91–7. doi: 10.1177/0891988706286512 16690994

[28] Ferghal M, Stephen H, Dean L, Karen M, Eimhear H, Sally P, et al. Acute neurological admissions in Northern Ireland: amount, hospital type and admission specialty using GIRFT methodology. In: Association of British neurologists: annual meeting abstracts 2023. BMJ Publishing Group Ltd; 2023. p. A92.1–A92. doi: 10.1136/jnnp-2023-abn.284

[29] McIntosh E, Kent S, Gray A, Clarke CE, Williams A, Jenkinson C, et al. Cost-effectiveness of dopamine agonists and monoamine oxidase B inhibitors in early Parkinson’s disease. Mov Disord. 2021;36(9):2136–43. doi: 10.1002/mds.28623 33960511

[30] Haycox A, Armand C, Murteira S, Cochran J, François C. Cost effectiveness of rasagiline and pramipexole as treatment strategies in early Parkinson’s disease in the UK setting: an economic Markov model evaluation. Drugs Aging. 2009;26(9):791–801. doi: 10.2165/11316770-000000000-00000 19728752

[31] McGregor P, O’Neill C. Resource allocation in the Northern Ireland health service: consensus or challenge?. Public Money & Management. 2014;34(6):409–16. doi: 10.1080/09540962.2014.962367

[32] Tosin MH, Goetz CG, Stebbins GT. Patient with parkinson disease and care partner perceptions of key domains affecting health-related quality of life: systematic review. Neurology. 2024;102(3):e208028. doi: 10.1212/WNL.0000000000208028 38215353 PMC11097757

